# Supplementary material for: Calorie restriction alters the mechanisms of radiation-induced mouse thymic lymphomagenesis
Source: PLoS One. 2023 Jan 20;18(1):e0280560. doi: 10.1371/journal.pone.0280560 (PMC9858762; doi:10.1371/journal.pone.0280560)
Supplement: S1 Fig — Names and locations of the microsatellite markers are shown in black, and representative tumor suppressor genes are shown in red. Dots indicate kinetochores. (DOCX) [file pone.0280560.s004.docx]

**
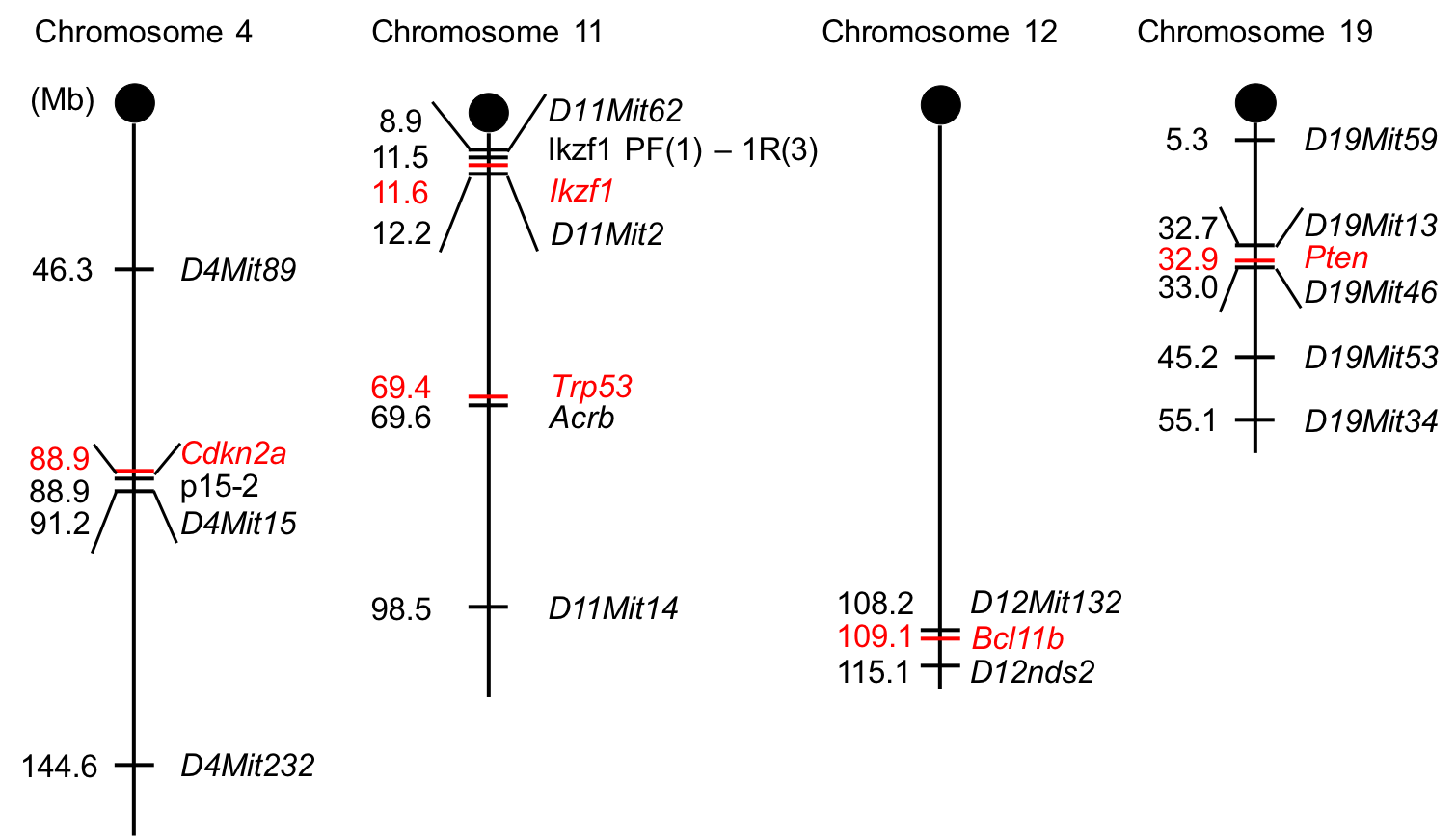
**

**S1 Fig.** Microsatellite markers used for the PCR-based LOH analysis. Names and locations of the microsatellite markers are shown in black, and representative tumor suppressor genes are shown in red. Dots indicate kinetochores.
