# Supplementary material for: Calorie restriction alters the mechanisms of radiation-induced mouse thymic lymphomagenesis
Source: PLoS One. 2023 Jan 20;18(1):e0280560. doi: 10.1371/journal.pone.0280560 (PMC9858762; doi:10.1371/journal.pone.0280560)
Supplement: S2 Fig — Left, diagram of chromosome 11, with the red bars indicating microsatellite markers to determine LOH. Right, DNA copy number analyzed by aCGH. The LOH was not detected by PCR because of its small size, residing between the nearest microsatellite markers. (DOCX) [file pone.0280560.s005.docx]

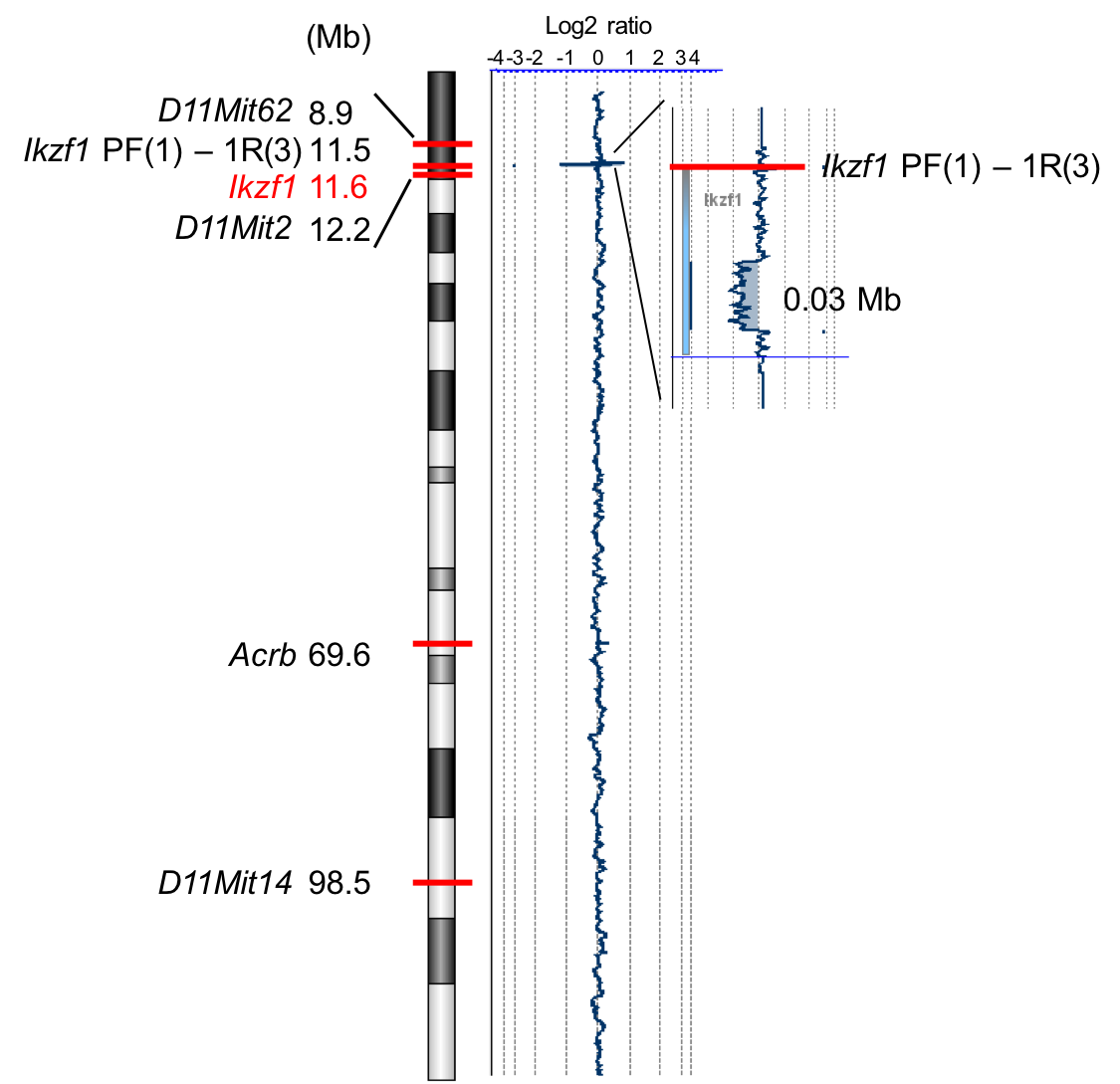


**S4 Fig.** An example of LOH that was detected by aCGH but not by PCR. Left, diagram of chromosome 11, with the red bars indicating microsatellite markers to determine LOH. Right, DNA copy number analyzed by aCGH. The LOH was not detected by PCR because of its small size, residing between the nearest microsatellite markers.
