# Supplementary material for: Calorie restriction alters the mechanisms of radiation-induced mouse thymic lymphomagenesis
Source: PLoS One. 2023 Jan 20;18(1):e0280560. doi: 10.1371/journal.pone.0280560 (PMC9858762; doi:10.1371/journal.pone.0280560)
Supplement: S3 Fig — Red bars, Pten locus. (A) A TL harboring a homozygous deletion formed via two different heterozygous deletions. (B) A TL harboring two copies of a deleted allele duplicated via mitotic recombination or mis-segregation. (DOCX) [file pone.0280560.s006.docx]

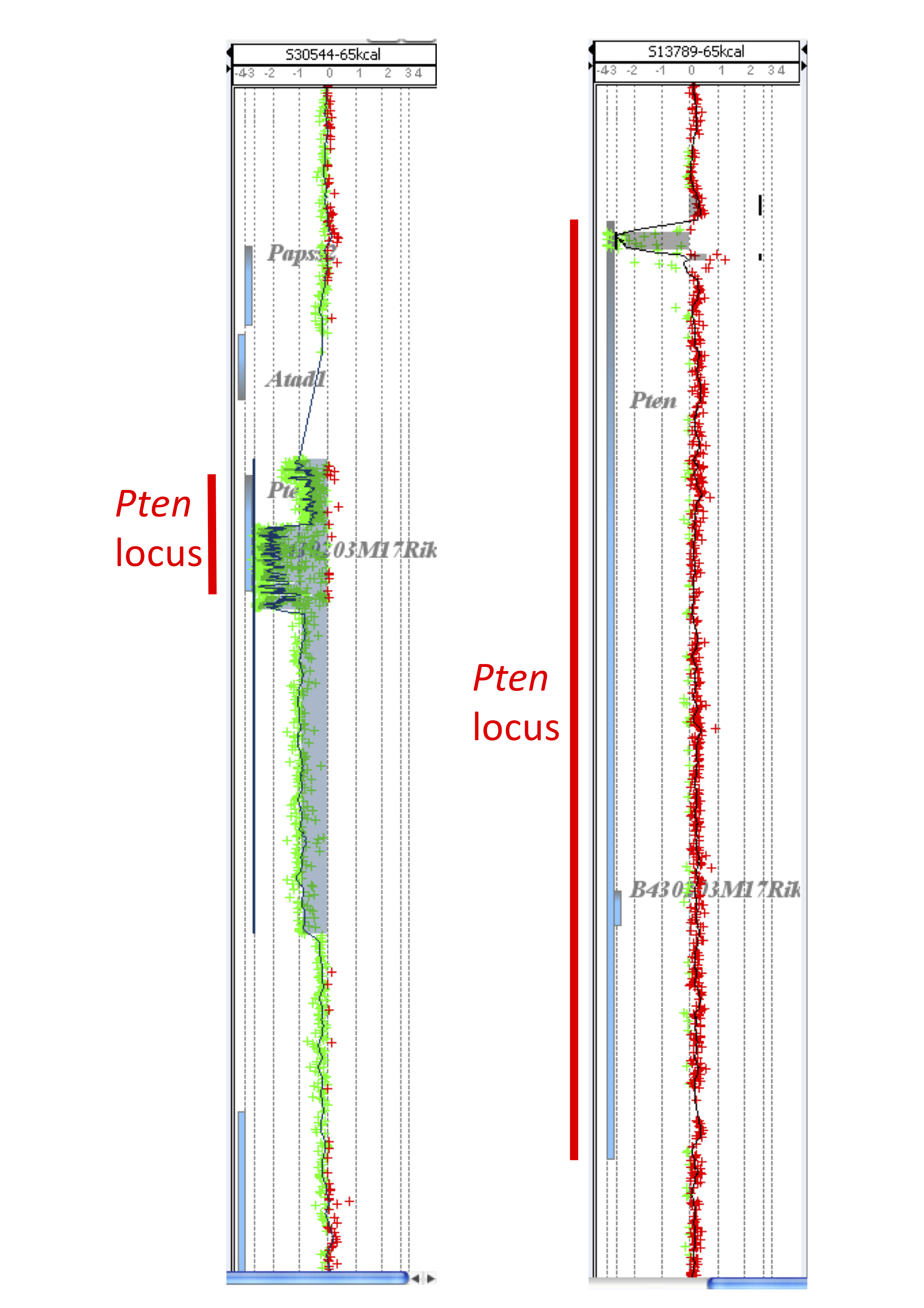


**S5 Fig.** Examples of homozygous deletions of *Pten*. Red bars, *Pten* locus. (A) A TL harboring a homozygous deletion formed via two different heterozygous deletions. (B) A TL harboring two copies of a deleted allele duplicated via mitotic recombination or mis-segregation.
