# Supplementary material for: Calorie restriction alters the mechanisms of radiation-induced mouse thymic lymphomagenesis
Source: PLoS One. 2023 Jan 20;18(1):e0280560. doi: 10.1371/journal.pone.0280560 (PMC9858762; doi:10.1371/journal.pone.0280560)
Supplement: S4 Fig — (A) Representative images of Ki-67 immunostaining. Brown, immunopositive cells; blue, counterstaining (hematoxylin). Scale bars, 1 mm. (B) Changes in the mean percentage of Ki-67+ cells in a tissue section over time. (C) Calculated weight of Ki-67+ cells in a thymus. Error bars, standard error (n = 3–6). (DOCX) [file pone.0280560.s007.docx]

**S6 Fig.** Cell proliferation in the thymus after CR was initiated. (A) Representative images of Ki-67 immunostaining. Brown, immunopositive cells; blue, counterstaining (hematoxylin). Scale bars, 1 mm. (B) Changes in the mean percentage of Ki-67**^+^** cells in a tissue section over time. (C) Calculated weight of Ki-67^+^ cells in a thymus. Error bars, standard error (*n* = 3–6).
