# Supplementary material for: Calorie restriction alters the mechanisms of radiation-induced mouse thymic lymphomagenesis
Source: PLoS One. 2023 Jan 20;18(1):e0280560. doi: 10.1371/journal.pone.0280560 (PMC9858762; doi:10.1371/journal.pone.0280560)
Supplement: S5 Fig — Chromosomes harboring trisomy in individual TLs are indicated by red boxes. (DOCX) [file pone.0280560.s008.docx]

**S8 Fig.** Trisomy in TLs. Chromosomes harboring trisomy in individual TLs are indicated by red boxes.
